# *In Situ* Investigation of the Phase Transition at the Surface of Thermoelectric PbTe with van der Waals Control

**DOI:** 10.34133/2022/9762401

**Published:** 2022-03-26

**Authors:** Feng Cheng, Ao Li, Siliang Wang, Yangjian Lin, Pengfei Nan, Shuai Wang, Ningyan Cheng, Yang Yue, Binghui Ge

**Affiliations:** Key Laboratory of Structure and Functional Regulation of Hybrid Materials of Ministry of Education, Institutes of Physical Science and Information Technology, Anhui University, Hefei 230601, China

## Abstract

The structure of thermoelectric materials largely determines the thermoelectric characteristics. Hence, a better understanding of the details of the structural transformation process/conditions can open doors for new applications. In this study, the structural transformation of PbTe (a typical thermoelectric material) is studied at the atomic scale, and both nucleation and growth are analyzed. We found that the phase transition mainly occurs at the surface of the material, and it is mainly determined by the surface energy and the degree of freedom the atoms have. After exposure to an electron beam and high temperature, high-density crystal-nuclei appear on the surface, which continue to grow into large particles. The particle formation is consistent with the known oriented-attachment growth mode. In addition, the geometric structure changes during the transformation process. The growth of nanoparticles is largely determined by the van der Waals force, due to which adjacent particles gradually move closer. During this movement, as the relative position of the particles changes, the direction of the interaction force changes too, which causes the particles to rotate by a certain angle.

## 1. Introduction

PbTe, a well-known thermoelectric material, is interesting to researchers because of its good thermoelectric properties and many potential applications [1–3]. It was previously reported that crystalline PbSe has low lattice thermal conductivity and can reach a high ZT value (>2) [4–6]. The excellent thermoelectric properties of PbTe may due to its structure: The highly symmetric crystal structure of PbTe enables high band-degeneracy and thus a significant effective mass. As a result, the lattice thermal conductivity can be maintained at a relatively low value [7].

However, many thermoelectric materials such as PbTe, SnTe, and PbSe have different phases, and because the different phases show different structures, the thermoelectric properties of the materials are affected directly [8–10].

Moreover, the transitions between different phase-structures of materials depend on environmental factors [11, 12]. To keep the structure stable and obtain excellent performance, both phase-transition conditions and processes were studied in depth. The trigger factors for phase-structure transformation include temperature, irradiation with an electron beam, and pressure [12–14]. In addition, the surface interface and dislocation defects also affect the phase-structure transition significantly [15–17].

Phase transitions in solids typically also include nucleation and growth [18, 19]. The main driving force for *nucleation* is a temperature gradient. The *growth process*, on the other hand, involves atomic motion, and its driving forces include temperature, van der Waals force, and so on. Compared to its interior, the atoms have a higher degree of freedom at the surface, which can provide more free space for the movement of atoms. This also means that significantly different phase-structure transformation processes may take place at the surface. Most studies of the PbTe phase transition mainly focused on the *internal* structure of the material [20–22], while the phase transition near the surface was rarely investigated. In addition, it is possible that surface state is closely related to their thermoelectric properties [23, 24], and the phase transition near the surface could play an important part of the whole phase-transition process.

In this study, highly crystalline PbTe was used (prepared). High-resolution transmission electron microscopy (HRTEM) with in situ heating was used to analyze the phase-transition process. By controlling both temperature and heating-time, the structural-transformation processes at the surface of solid PbTe could be observed with high accuracy. In addition, any occurring nucleation and growth mechanisms were revealed, during which the van der Waals force played an important role.

## 2. Results and Discussion

Both the structural characterization and the in-situ heating experiments were carried out using a JEOL JEM-F200 transmission electron microscope, which was operated at 200 kV. In situ heating of PbTe was performed with a DENS solutions-DH30-4M-JU and a double-tilt heating/biasing holder (chip: wildfire DS2049-W3-R1). The samples were fabricated with a Zeiss cross beam 550L system, which was equipped with a focused ion beam. Prior to ion-beam milling, an amorphous Pt-layer was deposited on the top surface to protect the films. During the in situ observation, the temperature was set to fixed values to keep the sample in a relatively stable state, and sequential HRTEM images were recorded using a Gatan RIO camera to track the structural changes during the heating process.

**Figure 1:**
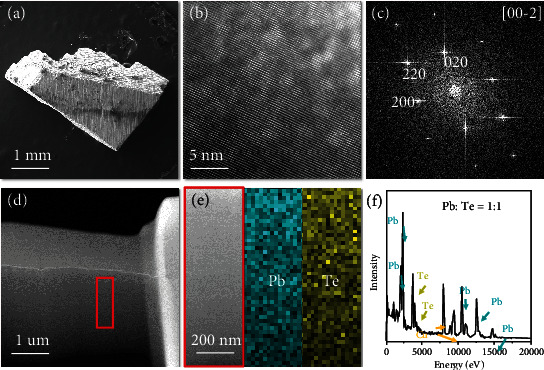
Morphology and crystal structure of PbTe. (a) SEM image of bulk PbTe. (b, c) HRTEM and corresponding FFT-images of PbTe. (d) HAADF image of PbTe. (e) The elemental maps for Pb and Te in PbTe show a uniform distribution of both elements. (f) EDS spectra of PbTe.

Figure 1(a) shows an SEM image of thermoelectric PbTe. The corresponding TEM images are shown in Figures 1(b)–1(d). The as-prepared PbTe (3-5 mm width) were uniform cubes with a face-centered cubic (fcc) structure and the space group *Fm-3m*. Furthermore, the crystal lattice constant (a ~0.65 nm) of PbTe, which was determined via Fast Fourier Transform (FFT) of the HRTEM image, was similar to values reported previously [25, 26]. The HAADF image (Figure 1(d)) shows that the highly crystalline material had no clear grain boundary, which indicates that the material is single crystalline. Color-coded elemental maps are shown in Figure 1(e). They show uniform distributions for Pb and Te. A more-detailed atomic-level element-distribution map is presented in Figure S1. It show an alternating arrangement of Pb and Te atoms, and the ratio of elements is always 1 : 1, which is confirmed by the obtained EDS spectra (Figure 1(f)).

**Figure 2:**
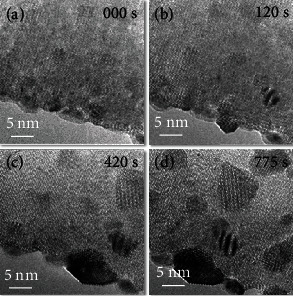
Phase transformation of PbTe at elevated temperature (400°C). (a–d) The sequential HRTEM images show the phase-transformation process at atomic resolution.

At high temperature (400°C) and when exposed to an electron beam (8000 e/Å2s), the structure of PbTe transformed.  Figure 2 shows that some PbTe restructured as well as new particles formed. The transformation processes triggered the formation of a crystal nucleus, and agglomeration occurred to produce larger particles. About 12 minutes later, many nanoparticles of different sizes formed. And the comparison of samples before and after heating is shown in Figure S2. An analysis of the structure and element information (Figure S3 and S4) showed that the formed particles did not show significant elemental changes and had an orthorhombic structure with the space group *Pnma* [27]. It was already reported that the structural transformation depends on surface defects, which is regarded as an important trigger factor for structural transformation [28]. We found that the structural transformation near the edges was faster than that in the center, which confirms the important function of the material surface during the structural transformation.

**Figure 3:**
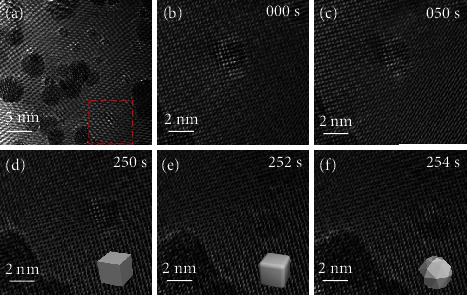
Images used to study the nucleation process and the geometric-structure transformation. (a) HRTEM image of PbTe (*Pnma*) particles and crystal nucleus at elevated temperature of 400°C. (b–f) Process of geometric-structure transformation of the PbTe crystal nucleus.

Because the transformation process also includes both nucleation and growth, these were also analyzed. As Figure S5 shows, the initial nucleation density of PbTe (*Pnma*) was very high, and many new nanoparticles (1-3 nm in size) formed gradually. Interestingly, the initial geometric structures of the formed crystal-nucleus were either cubic or rectangular. As the volume of the particle increased, its shape gradually transformed into a polyhedron. It was reported that in a critical crystal nucleus, the geometry of the structure is closely related to size, and different geometries may lead to different energies in the material [29, 30]. After analyzing both the transformation of the material geometry and the growth process of the material, we found that both processes occurred during heating. To clarify the priorities of the geometric-structure transformation and the material growth process, both the corresponding specific in situ geometric-structure-transformation process for PbTe (*Pnma*) and the nucleus size were investigated (see Figure 3).

Figure 3 shows the geometric structure of transformed PbTe (*Pnma*). The geometric shape of the particles in Figure 3(b) shows clear differences from Figure 3(f). This indicates that the geometric-structure transformation may occur prior to material growth. In addition, the transformation of the geometric structure occurs practically instantly. Figures 3(d)–3(f) (movie S1) show that the transformation process takes place within five seconds, and the geometry of the particles remains unchanged before this. This may be due to the energy barrier because it was reported that the transformation of the material structure needs to overcome a certain energy barrier [31, 32]. With increasing time, when the accumulated energy exceeds the energy barrier needed for the structural transition, the reaction starts rapidly.

**Figure 4:**
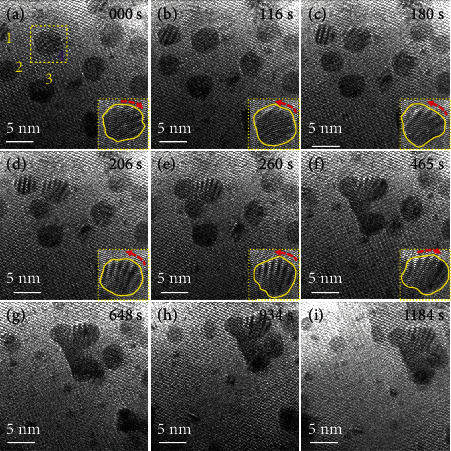
Movement process of nanoparticles during the phase transition at elevated temperature, 400°C. (a–i) HRTEM images of the nanoparticles during movement as a function of time. The lower insets are corresponding magnifications to clarify the change in morphology and the angle for nanoparticle 4.

To analyze the growth process of the phase transformation, sequences of HRTEM images were recorded (see Figure 4). The aggregation and growth processes of the particles can be seen clearly. As Figure 4 shows, particles 1-4 appeared relatively independent, and at high temperature, the particles interact with each other and finally create large particles. Furthermore, the particles showed a significant angular deflection during the growth process. This is similar to crystal growth in the so-called “oriented-attachment” growth mode [33, 34]. Figures 4(d)–4(f) indicate that particles 2 and 4 contacted to form the initial physical interface. As a result, a neck (Figure S6) appears. Subsequently, the particles grew into larger particles. In order to better confirm the growth process of particles and exclude the influence of substrate on image definition, more clear images of similar particles in edge region is shown in Figure S7, and the corresponding growth mode is also well matched with oriented attachment.

According to published reports of oriented attachment, rotation occurs after the particles contacted each other and formed a neck. However, we found that crystal particles also showed deflection at a certain angle before contacting occurred. Figures 4(a) and 4(b) (movie 2) show that the nanoparticles rotated in different directions, and their movement speed increased with decreasing distance. This may be closely related to the van der Waals force. It was already reported that, within a certain range, the van der Waals force increases with decreasing distance [35, 36], and the increasing interaction forces increase the speed of moving particles. The change of the relative position of the particles may lead to a change in the direction of the van der Waals force, which may also change the movement direction of the nanoparticles. In addition, since the van der Waals forces between the two particles are the same, due to the different sizes, the movement distance of the particles is different. This is confirmed by the experimental results. The movement distance of the larger particle 4 was smaller than the particles 1-3. To avoid contingency and the size effect, particles of different sizes were observed. Figure S8 shows that the smaller particles (which have a similar size as the crystal nucleus) show a similar phenomenon, close to the large particles.

The phase-structure transformation can occur in the bulk or on the surface of the material. Because the TEM image is a projection, it was difficult to determine the specific position of the phase transition when the two structures overlap. To clarify the region of the phase structure transformation, we increased the temperature (500°C) and electron beam intensity (20000 e/Å^2^s), which can separate the two structures. Figure S9 shows that, due to the high energy, the substrate gradually disappeared, and particles appeared gradually. Furthermore, the high-resolution image of the boundary shows that both the particles and the substrate were at different heights, and the particles were mainly attached to the surface of the substrate via adsorption. To further confirm this result, the HAADF images are acquired at different magnification (Figure S10), and the results are also well matched. In other words, the phase transformation mainly occurred on the material surface. During the transformation, the main driving forces are temperature and electron beam irradiation; the high spatial freedom of atoms at the surface provides favorable conditions to facilitate the movement of particles.

At the same time, in order to clarify the role of temperature and electron beam irradiation, the controlled experiments are shown in Figure S11 and S12. As Figure S11 shows, with low electron beam intensity, the new particles formed and growthed normally. With the increase of the electron beam intensity, the material in the region with high electron beam irradiation restructured more obvious (Figure S11a). According to the report that the temperature will directly affect by the electron beam [37], we think that the electron beam irradiation may rise the local temperature and make the phase transition easy to start. To estimate the temperature induced by electron beam heating, the corresponding calculation is done (S9), and the results show that the temperature rise induced by electron beam heating with dose rate of 8000 e/Å^2^s should be less than 60°C.

## 3. Conclusion

In summary, thermoelectric PbTe was successfully prepared, and the temperature dependence of the phase transition was directly observed using in situ TEM. After exposure to an electron beam as well as increased temperature, the structure of PbTe changed form face-centered cubic to orthorhombic. During the process, because of the high temperature and electron beam, high-density crystal nuclei (with size of about 1-3 nm) formed. The geometric structure of the formed crystal nuclei was closely related to their size. The initial geometric structure of the formed crystal nucleus was either cubic or rectangular and then changed to polyhedron. The formed particles grew in accordance with the known oriented-attachment growth mode, and they changed their geometric structure. The formation of the particles was directly affected by the van der Waals force, due to which the adjacent particles get closer to each other and rotate with a certain angle. The results also show that the phase transition mainly occurred on the surface of the material—possibly a consequence of the high surface-energy of the material. The results would provide valuable information for studying the surface structure transformation of thermoelectric materials and are inspiring for further understanding the factors affecting surface stability, as well as providing a new idea for surface engineering of thermoelectric materials.

## Data Availability

The data used to support the findings of this study are available from the corresponding author upon request.

## Supplementary Materials

Supplementary Materials Figure S1: HAADF images and atomic level element distribution map. Figure S2: comparison of samples before and after heating. Figure S3: PbTe particles with structure of orthorhombic. Figure S4: element information of the restructured samples. Figure S5: the TEM images of PbTe of different regions at elevated temperature. Figure S6: the TEM image of PbTe, in which the two particles are connected by a “neck.” Figure S7: the evolution of the PbTe particles under continuous heating at 400°C. Figure S8: the growth process of the PbTe particles with the comparison of size effect. Figure S9: the analysis of the relative position between the substrate and formed particles. Figure S10: the HAADF images of the relative position between the substrate and formed particles. Figure S11: nucleation and growth during phase transition of PbTe at elevated temperature (400°C) with low electron beam intensity. Figure S12: the investigation of the effects of the electron beam irradiation. Movie 1: process of geometric-structure transformation of the crystal nucleus at elevated temperature of 400°C. Movie 2: movement process of nanoparticles during the phase transition at elevated temperature of 400°C.
