# Supplementary material for: In Situ Investigation of the Phase Transition at the Surface of Thermoelectric PbTe with van der Waals Control
Source: Research (Wash D C). 2022 Mar 26;2022:9762401. doi: 10.34133/2022/9762401 (PMC8978022; doi:10.34133/2022/9762401)
Supplement: Supplementary Materials — Figure S1: HAADF images and atomic level element distribution map. Figure S2: comparison of samples before and after heating. Figure S3: PbTe particles with structure of orthorhombic. Figure S4: element information of the restructured samples. Figure S5: the TEM images of PbTe of different regions at elevated temperature. Figure S6: the TEM image of PbTe, in which the two particles are connected by a “neck.” Figure S7: the evolution of the PbTe particles under continuous heating at 400°C. Figure S8: the growth process of the PbTe particles with the comparison of size effect. Figure S9: the analysis of the relative position between the substrate and formed particles. Figure S10: the HAADF images of the relative position between the substrate and formed particles. Figure S11: nucleation and growth during phase transition of PbTe at elevated temperature (400°C) with low electron beam intensity. Figure S12: the investigation of the effects of the electron beam irradiation. Movie 1: process of geometric-structure transformation of the crystal nucleus at elevated temperature of 400°C. Movie 2: movement process of nanoparticles during the phase transition at elevated temperature of 400°C. [file 9762401.f1.zip › Supporting-Information.docx]

**Supporting Information**

***In-Situ* Investigation of the Phase Transition at the Surface of Thermoelectric PbTe with Van Der Waals Control**

*Feng Cheng*, Ao Li, Siliang Wang, Yangjian Lin, Pengfei Nan, Shuai Wang, Ningyan Cheng, Yang yue*, Binghui Ge**

Dr. F. Cheng, A. Li, Dr. S. Wang, Y. Lin, P. Nan, S. Wang, Dr. N. Cheng, Dr. Y. Yue, Prof. B. Ge

Key Laboratory of Structure and Functional Regulation of Hybrid Materials of Ministry of Education, Institutes of Physical Science and Information Technology

Anhui University, 111 Jiulong Road, Hefei, Anhui 230601, China

1. mail: [chengfeng769@126.com; yueyang@ahu.edu.cn; bhge@ahu.edu.cn.](mailto:luying.li@hust.edu.cn,)
2. **Synthesis of PbTe single crystaline**
3. **HAADF images and EDS mapping**
4. **Structural analysis and element information of the formed particles**
5. **Analysis of PbTe nucleation**
6. **Formation of the ‘neck’ and the growth process of PbTe particles following oriented attachment modle**
7. **Growth process of the PbTe particles with small size**
8. **Analysis of the relative position between the base and formed particles.**
9. **Analysis of the effects of the electron beam irradiation.**
10. **Estimation of temperature induced by electron beam heating.**
11. **Synthesis of PbTe single crystaline**

Single crystalline PbTe samples were prepared by Te-flux method with the high purity elements of Pb (grains, 99.99%) and Te (grains, 99.999%) as starting materials, which were weighed according to the stoichiometric ratio of Pb:Te = 1:4. The mixture was sealed in an evacuated quartz tube and heated to 1173 K in 3 h. After holding at this temperature for 20 h, the furnace slowly cooled to 723 K at a cooling rate of 13 K/h. Subsequently, single crystalline PbTe samples were separated from the molten Te-flux by centrifugation technology.

1. **HAADF images and EDS mapping**
2.
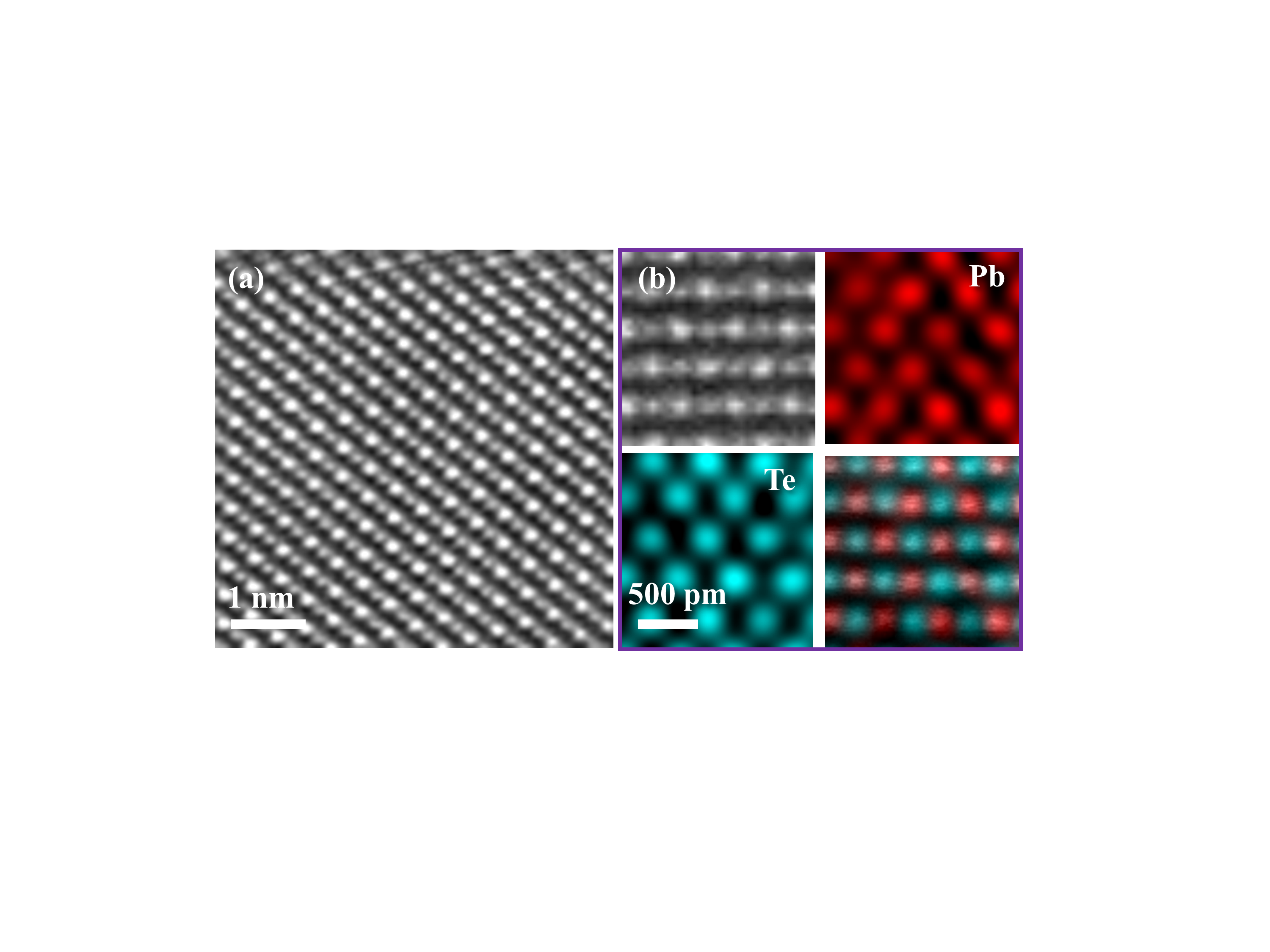


**Figure S1**. HAADF images and atomic level element distribution map. (a) The HAADF image of PbTe. (b) The element distribution of Pb and Te at atomic level.

1. **Structural analysis and** **element information of the formed particles**


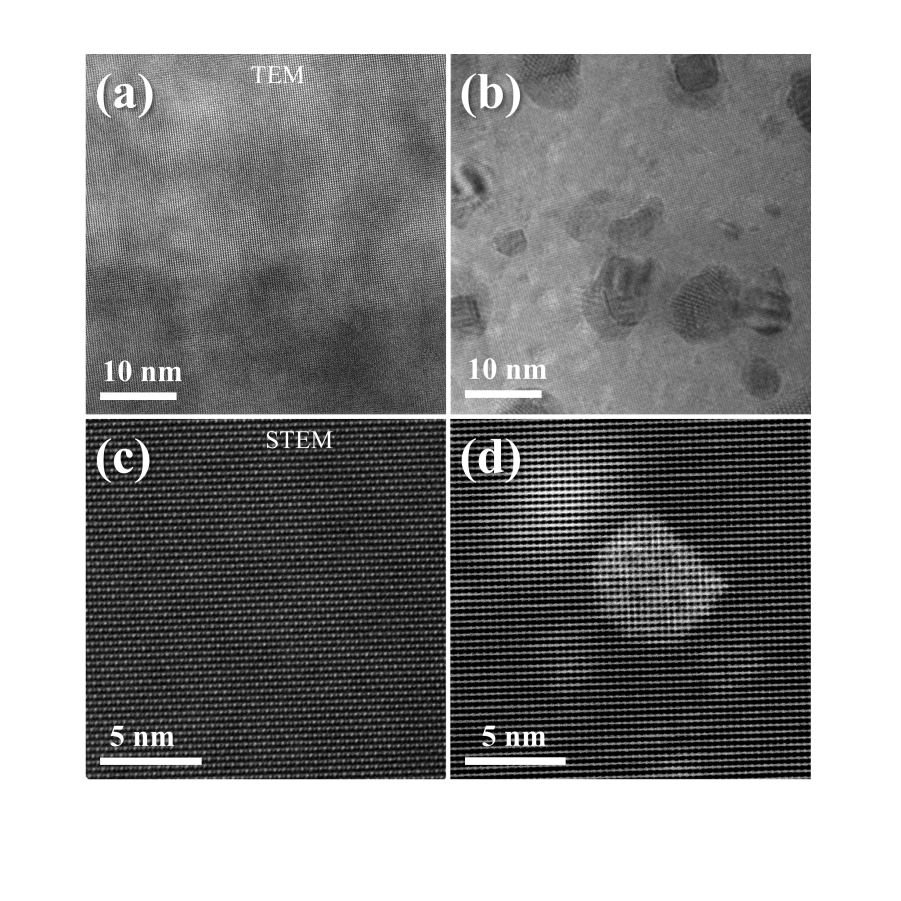


Figure S2 comparison of samples before and after heating. (a) (c) The TEM and STEM images of the samples before heating. (b) (d) The TEM and STEM images after heating at elevated temperature of 400°C.


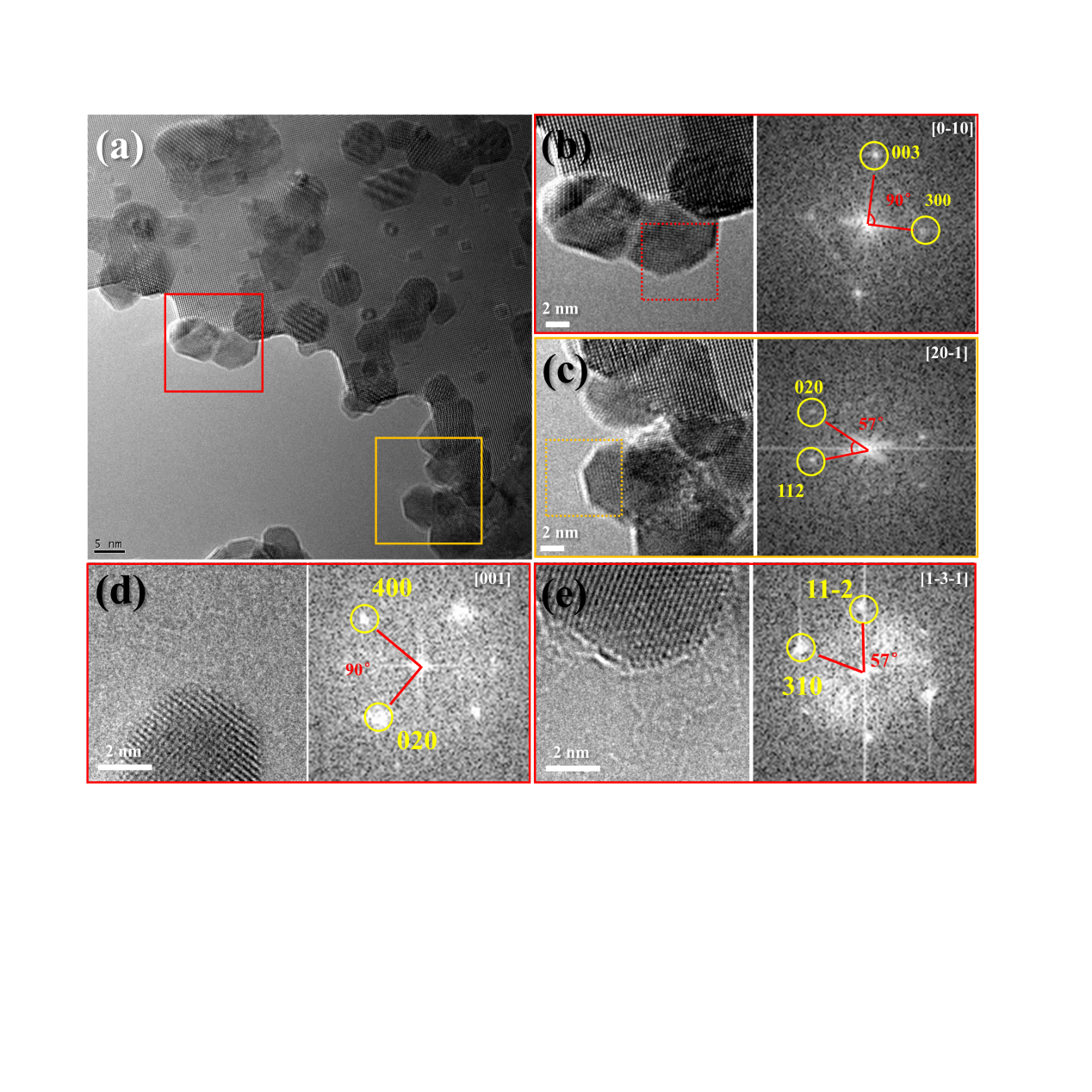


**Figure S3**. PbTe particles with structure of orthorhombic. (a) TEM image of the particles at elevated temperature of 400°C. (b, c) The crystal information of the part of formated particles in image a. (d, e) The crystal information of particles in other similar region.


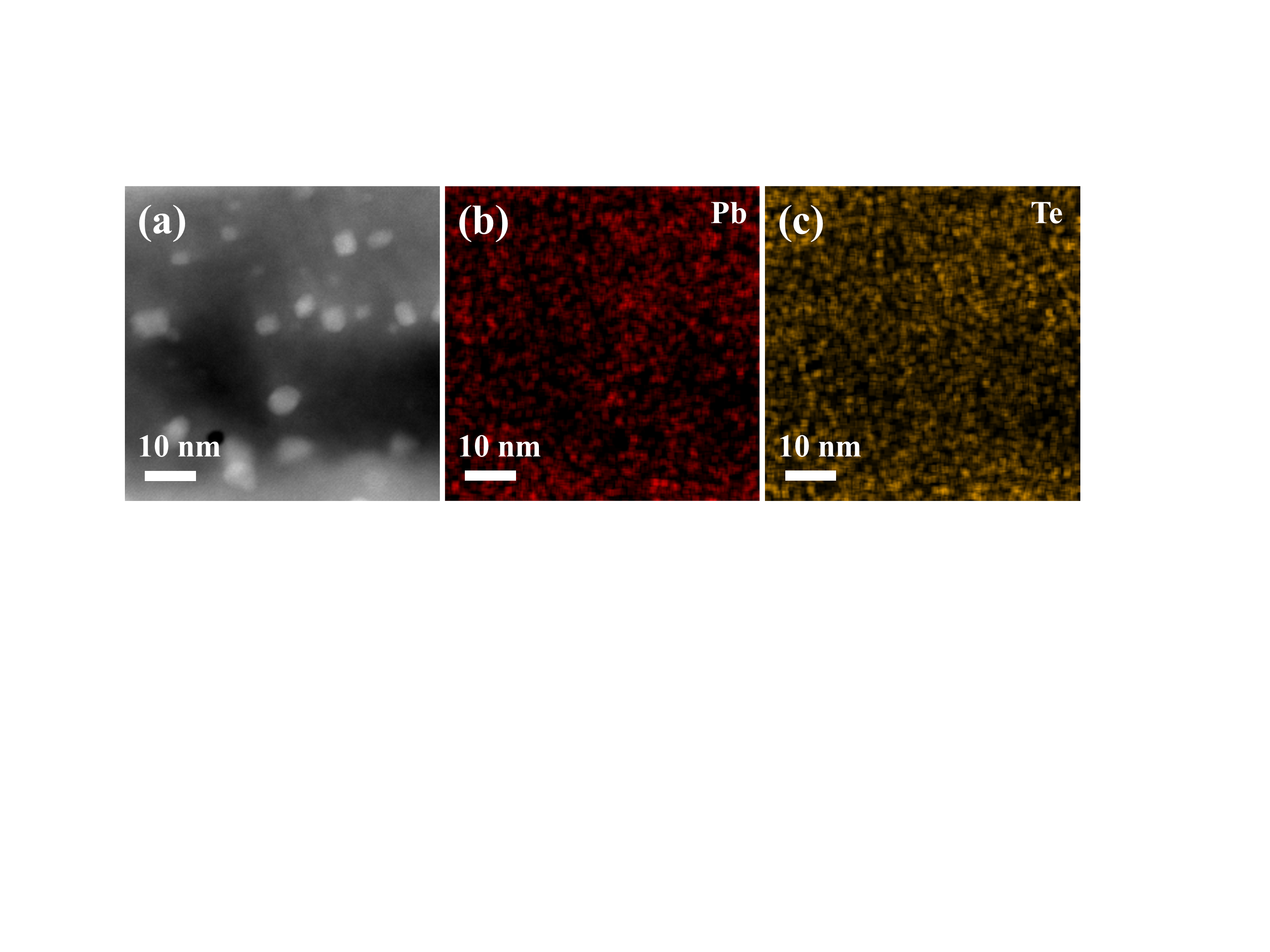


**Figure S4**. Element information of the restructured samples. (a) HAADF image of PbTe. (b-c) The corresponding elemental maps of Pb and Te.

1. **Analysis of PbTe nucleation**


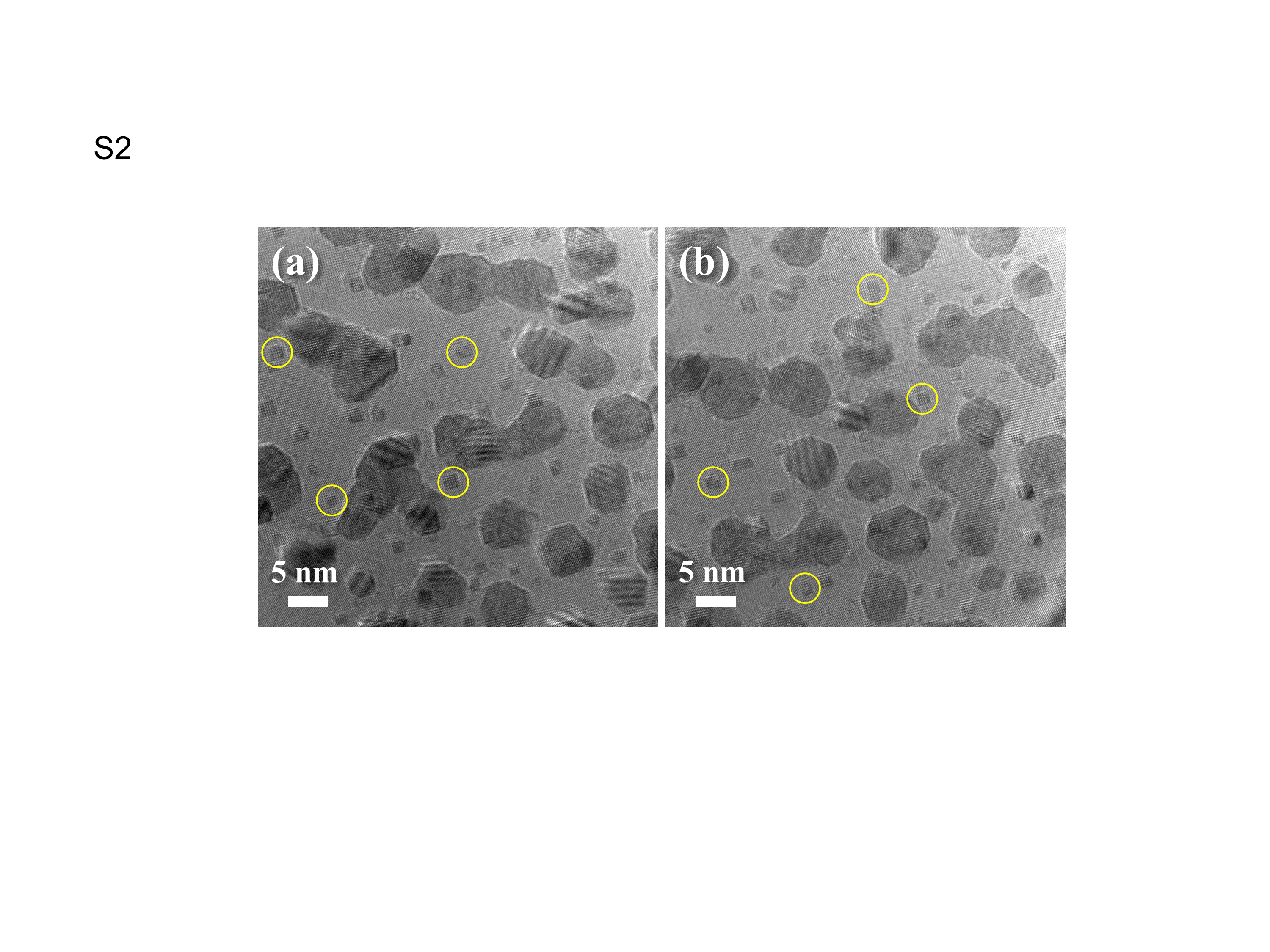


**Figure S5**. The TEM images of PbTe of different regions at elevated temperature of 400°C after 10 minutes.

1. **Formation of the ‘neck’ and the growth process of PbTe particles following oriented attachment modle.**


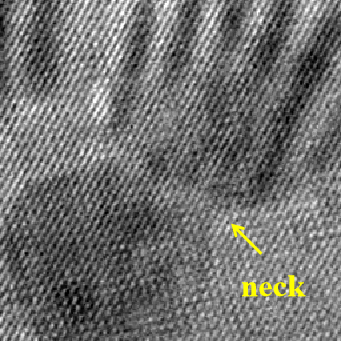


**Figure S6**. The TEM image of PbTe, in which the two particles are connected by a ‘neck’.


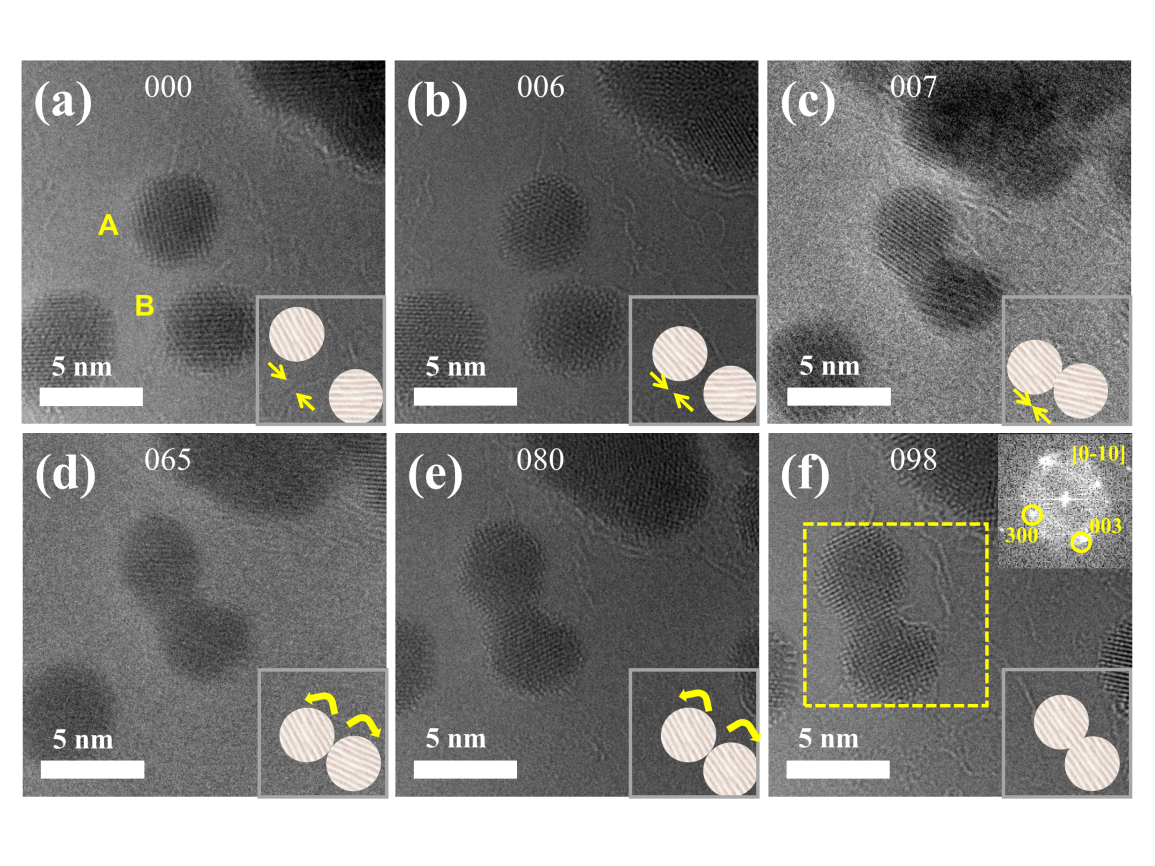


**Figure S7**. The evolution of the PbTe particles under continuous heating at 400 °C. (a-f) The HRTEM images of the PbTe particles with different growth time at 400 °C, and the illustrations is the corresponding sketches of the evolution of PbTe particles. According to which, the growth modle is well matched with oriented attachment.

1. **Growth process of the PbTe particles with small size**


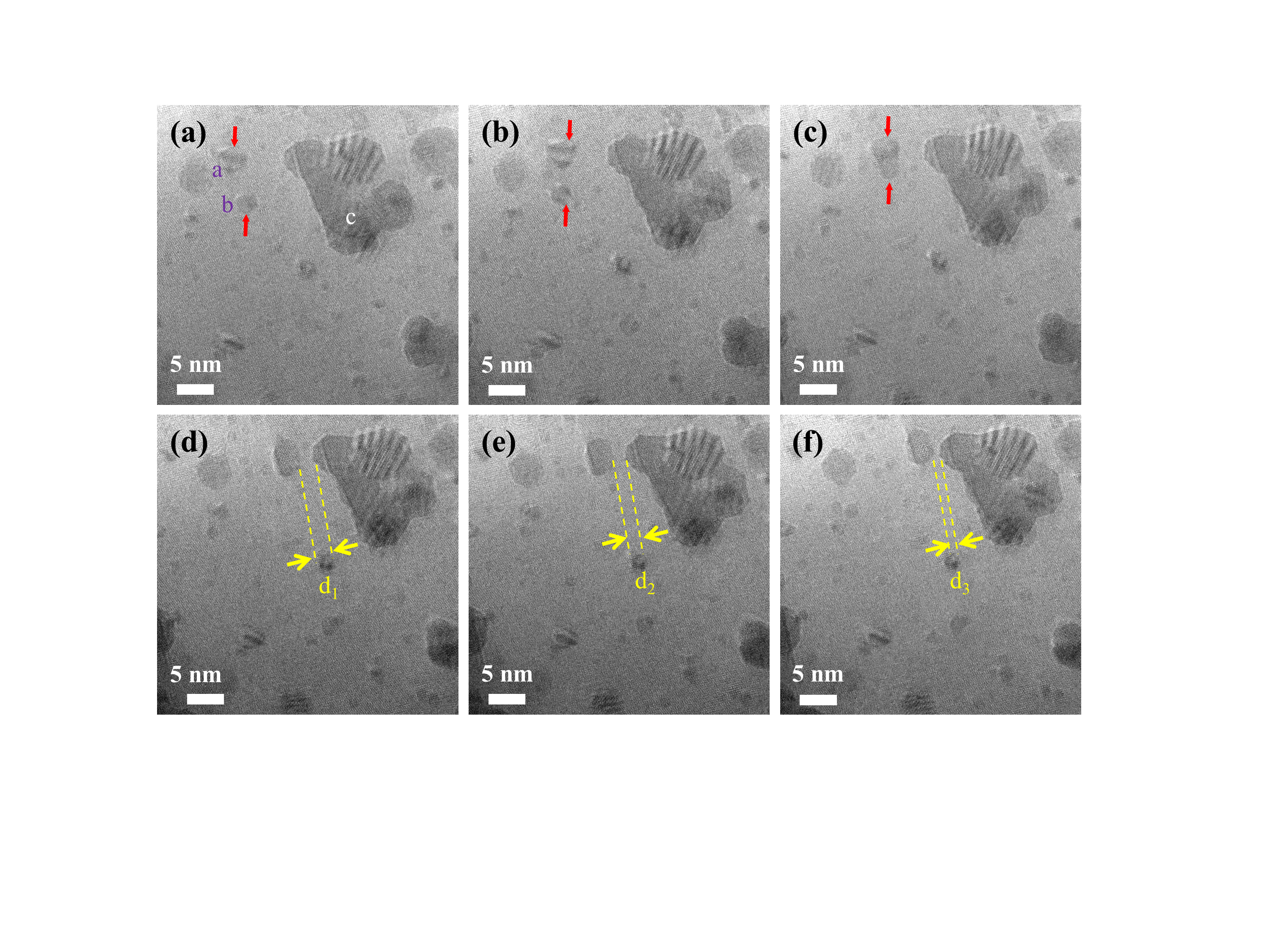


**Figure S8**. The growth process of the PbTe particles with the comparison of size effect. (a-c) The fusion process of particles a and b. (f-g) The fusion process of particles a and c. The particles a and b used to show a small size of 2-3nm, and during the fusion processes, the relative positions of the particles a b and c changed, which used to form a larger particle.

1. **Analysis of the relative position between the base and formed particles.**


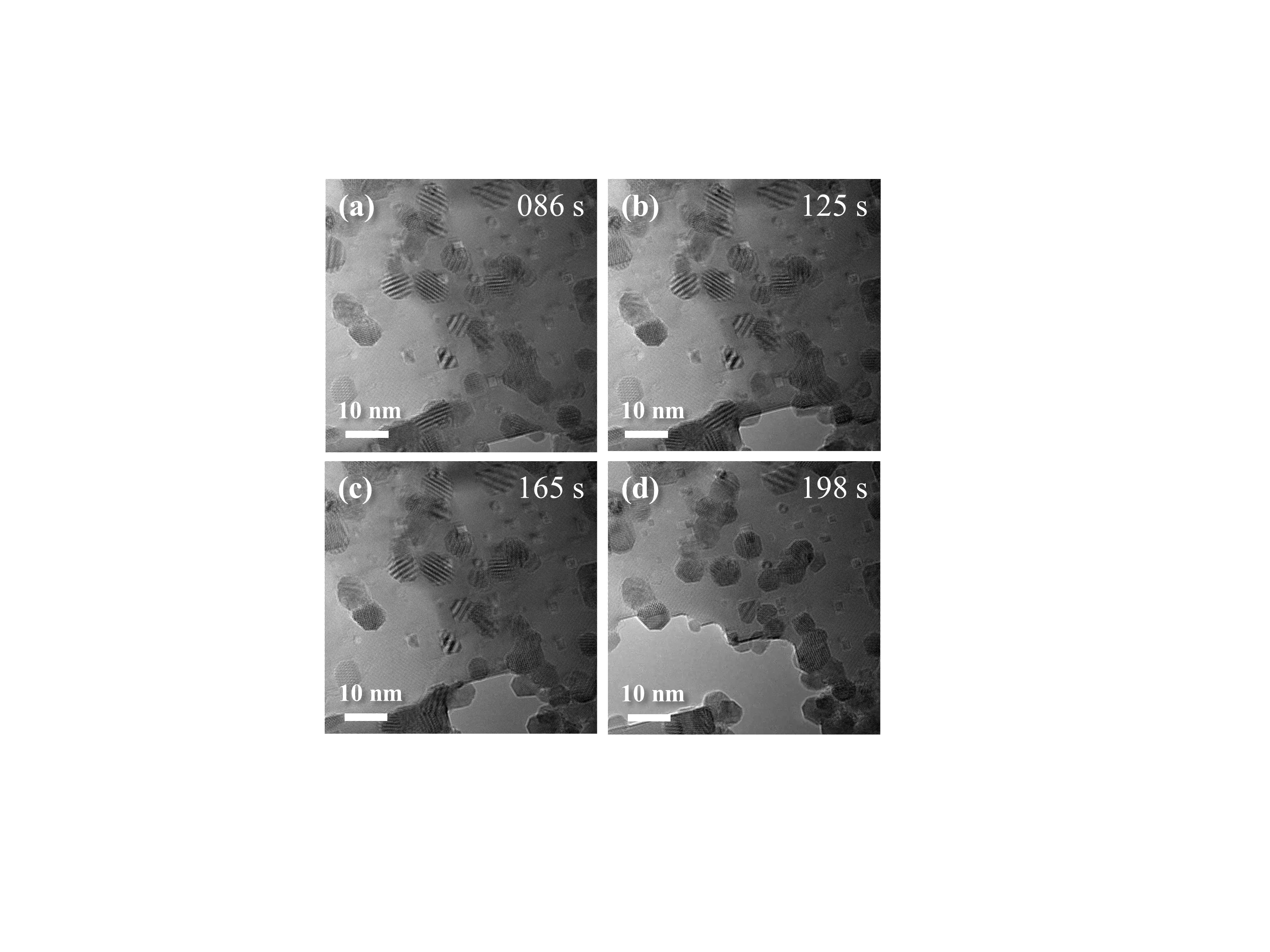


**Figure S9**. The analysis of the relative position between the substrate and formed particles. (a-d) The TEM images of the PbTe with the electron beam intensity of 20000 e/Å^2^s at elevated temperature of 500°C. Due to high temperature and electron beam intensity the substrate gradually disappears, and which show a relative position of that the formed particles mainly on the surface of the substrate.


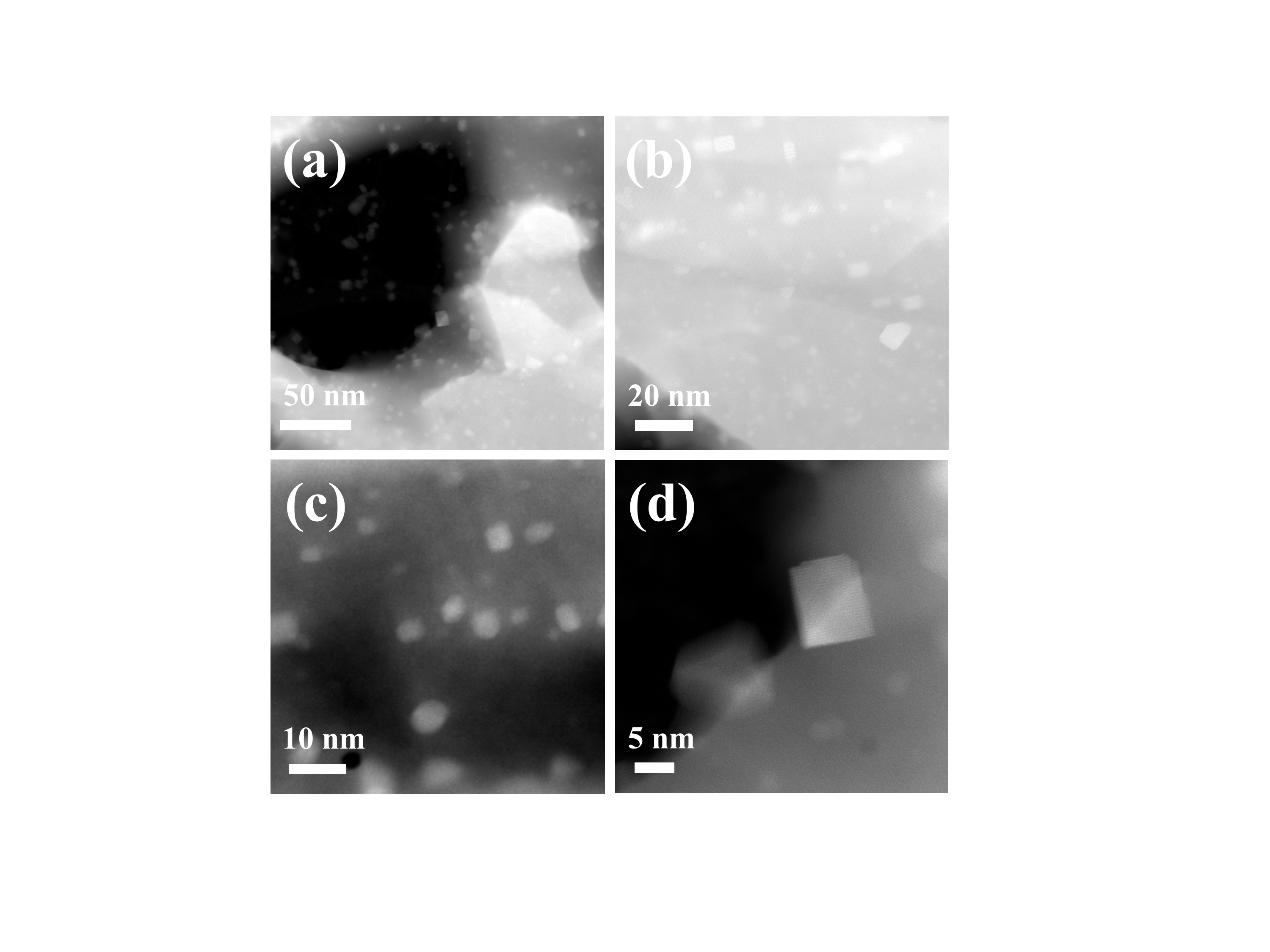


Figure S10. The HAADF images of the relative position between the substrate and formed particles. (a-d) The HAADF images with different magnification at 400 °C, from which it can clear shows that the formed particles are on the surface of the substrate.

1. **Analysis of the effects of the electron beam irradiation.**


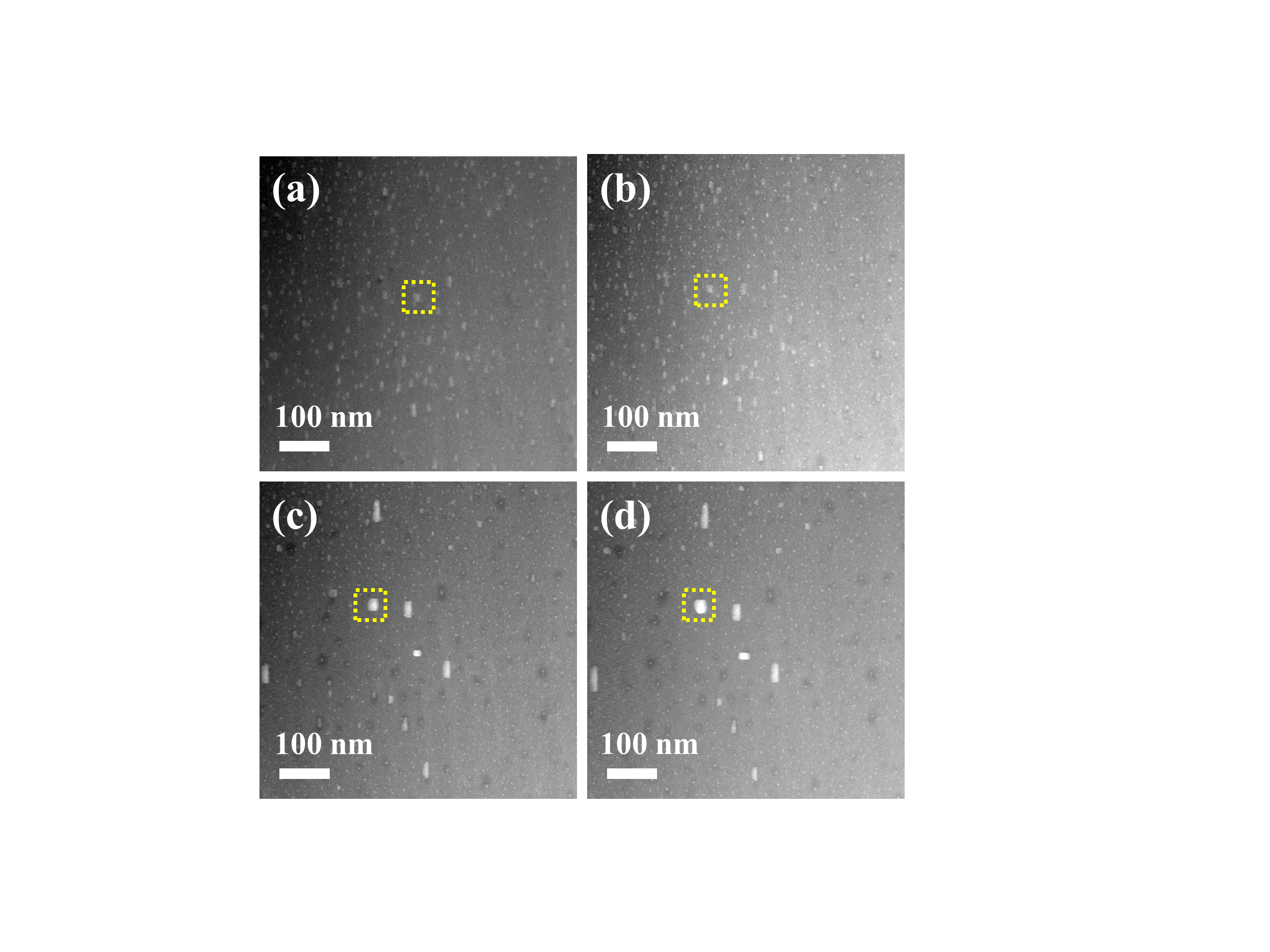


Figure S11. Nucleation and growth during phase transition of PbTe at elevated temperature (400 °C) with low electron beam intensity. (a-d) The sequential HAADF images show the processes of the nucleation and growth.


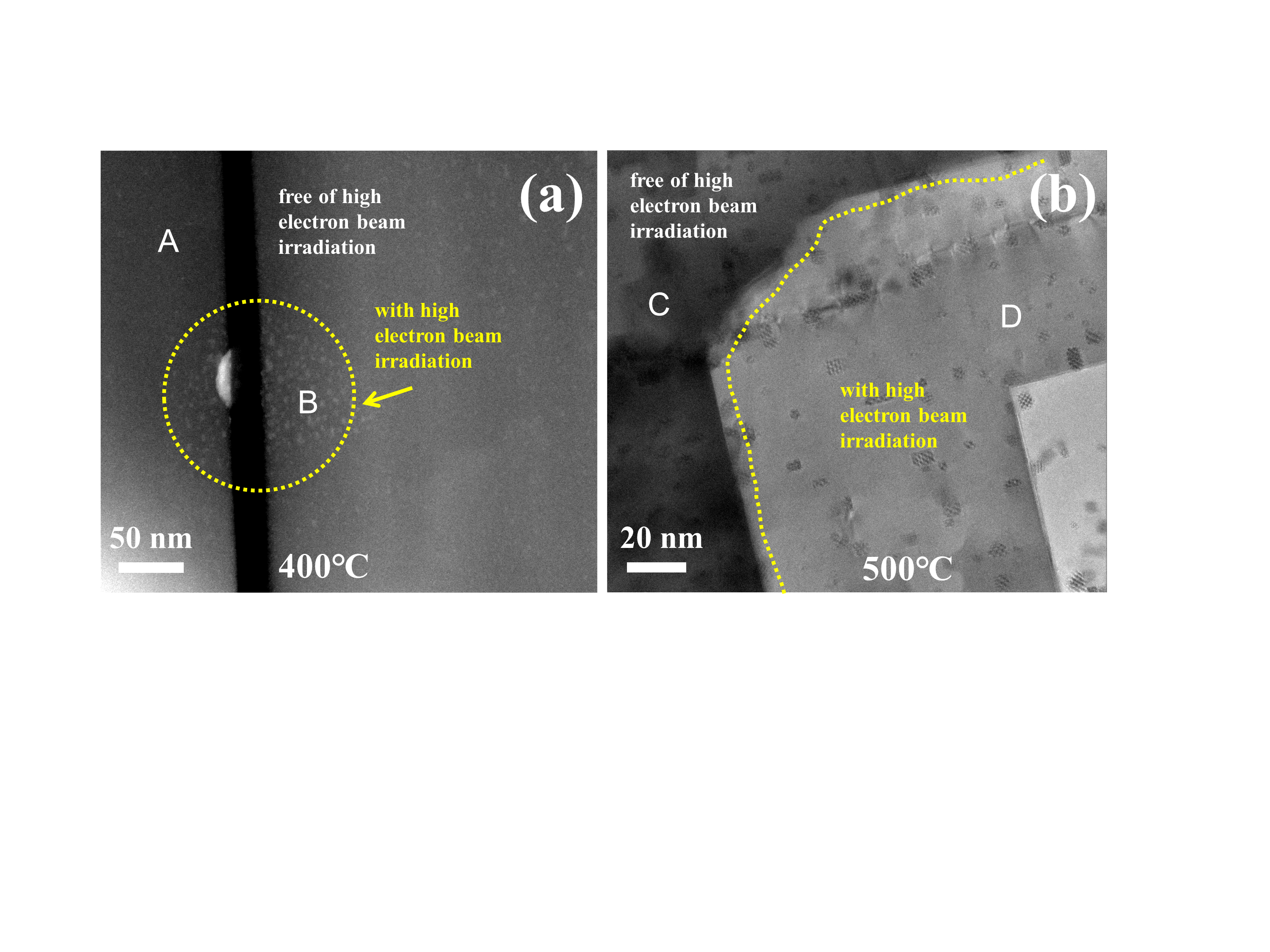


Figure S12. The investigation of the effects of the electron beam irradiation. (a) The HAADF image of the PbTe after heating for five minutes at 400 °C, the region B is irradiated by electron beam for five minutes with the electron beam intensity of 8000 e/Å^2^s, the region A is free of electron beam irradiation. (b) The TEM image of the PbTe after heating for one minutes at 500 °C, the region D is irradiated by electron beam for one minute with the electron beam intensity of 20000 e/Å^2^s, the region C is free of electron beam irradiation. Compare with the region A and B, it can be seen that the phase transition is easy to start in region B.

1. **Estimation of temperature induced by electron beam heating.**

The temperature increase under irradiation is calculated by Fisher’s mode^[1]^:

$$\Delta T=\frac{I}{\pi ke}(\frac{\Delta E}{d})ln\frac{b}{r_{0}}$$

where *I* is the beam current, *k* is the thermal conductivity, *e* is the electron charge, *b* is the sample radius, *r_0_* is the beam radius, and ∆*E* is the total energy loss per electron in a sample with a thickness of *d*. Because energy losses in the sample are small compared to the initial energy of electrons (200 keV), the term ∆*E*/*d* equals to the stopping power of electrons, d*E/*d*x*, which can be calculated from the Bethe-Bloch equation:

$$-\frac{dE}{dx}=\frac{2\pi Zp(e^{2}/4\pi\varepsilon_{0}^{2})^{2}}{mv^{2}}\{ln[\frac{E(E+mc^{2})\beta^{2}}{2I_{e}^{2}mc^{2}}]+(1-\beta^{2})-(1-\sqrt{1-\beta^{2}}+\beta^{2})ln2+\frac{1}{8}(1-\sqrt{1-\beta^{2}})^{2}$$

where *Z* is the atomic number of the target element, *ρ* is the atomic density, *ε_0_* is the

dielectric constant, *m* is the electron rest mass, *v* is the electron velocity, *c* is the speed

of light, *E* is the electron energy, *I_e_* is the average excitation energy of electrons in the

target, and *β = v/c*.

In the present study, the acceleration voltage is 200 kV. Thus, *β =* 0.70 and v = 2.10 × 10^8^ m/s. For Pb and Te, Z_Pb_ = 82, Z_Se_ = 52. As Z_Pb_ » Z_Te_, we use only Pb atoms to represent PbTe atoms in this calculation. *I_e_* = 8.8Z = 721.6, *m* = 9.3×10^-31^kg, *ρ* = 2.98×10^28^ m^-3^, *e* = 1.6×10^-18^, thermal conductivity *k* = 1.1 W/mK^[2]^, dielectric constant *ε_0_* = 3.54 ×10^-9^ F/m^[3]^. The maximum temperature increase is estimated to be less than 60 ^o^C with electron beam density of 8000 e/Å^2^S at 400 ^o^C.

1. Ic, I. J.; Bench, M. W.; Robertson, I. M.; Kirk, M. A. Electron‐beam‐induced crystallization of isolated amorphous regions in Si, Ge, Gap, and Gaas. *J. Appl. Phys.* **1995**, 78, 974-982.

(2) Biswas, K.; He, J.; Wang, G.; Lo, S. H.; Uher, C.; Dravid, V. P.; [Kanatzidis](https://xueshu.baidu.com/s?wd=author:(Mercouri G. Kanatzidis) Department of Chemistry&tn=SE_baiduxueshu_c1gjeupa&ie=utf-8&sc_f_para=sc_hilight=person" \t "https://xueshu.baidu.com/usercenter/paper/_blank), M.G. High thermoelectric figure of merit in nanostructured p-type PbTe–mte (m = ca, ba). *Energy Environ. Sci.* **2011**, 4, 4675-4684.

(3) Kanai, Y.; Shohno, K. Dielectric constant of PbTe. *Jpn. J. Appl. Phys.* **1963**, 2, 6-10.
